# A qualitative exploration of the facilitators and barriers to self-management in kidney transplant recipients

**DOI:** 10.1007/s40620-022-01325-w

**Published:** 2022-04-25

**Authors:** Katherine E. Memory, Thomas J. Wilkinson, Alice C. Smith, Courtney J. Lightfoot

**Affiliations:** 1 Leicester Medical School, University of Leicester, Leicester, UK; 2 Leicester Kidney Lifestyle Team, Department of Health Sciences, University of Leicester, Leicester, UK

**Keywords:** Transplantation, Kidney transplant recipients, Self-management, Patient activation

## Abstract

**Background:**

Understanding the behaviours that facilitate or impede one’s ability to self-manage is important to improve health-related outcomes in kidney transplant recipients (KTRs). Previous studies exploring the self-management experiences of KTRs have focused on specific tasks (e.g., medication adherence), age groups (e.g., adolescent or older recipients), or have been conducted outside of the UK where transferability of findings is unknown. Our study aimed to explore the perceptions and experiences of self-management in UK KTRs to identify facilitators and barriers associated with self-management tasks.

**Methods:**

Semi-structured interviews were conducted with eleven KTRs. Topics explored included experiences of self-management tasks (diet, exercise, medications, stress management), perceived healthcare role, and future interventional approaches. Thematic analysis was used to identify and report themes.

**Results:**

Eight themes were identified which were mapped onto the three self-management tasks described by Corbin and Strauss: medical, role and emotional management. Perceived facilitators to self-management were: gathering health-related knowledge, building relationships with healthcare professionals, creating routines within daily life, setting goals and identifying motivators, establishing support networks, and support from family and friends. Complexity of required treatment and adjusting to a new health status were perceived barriers to self-management.

**Conclusions:**

Participants described the importance of collaborative consultations and continuity of care. Tailored interventions should identify individualised goals and motivators for participating in self-management. Education on effective strategies to manage symptoms and comorbidities could help alleviate KTRs’ perceived treatment burden. Family and peer support could emotionally support KTRs; however, managing the emotional burden of transplantation warrants more attention.

**Graphic abstract:**

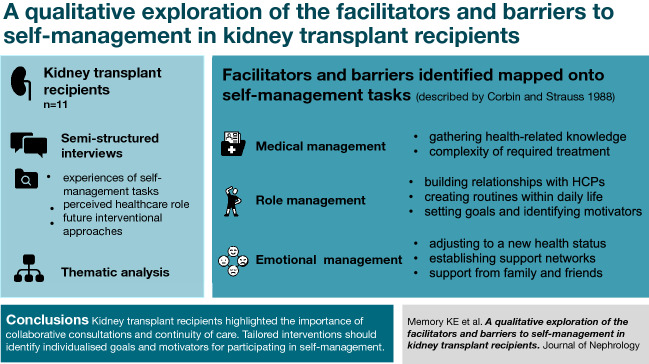

## Introduction

Kidney transplantation offers better long-term health outcomes [1, 2] and reduced mortality [3] than dialysis for individuals with end-stage kidney disease. However, morbidity and quality of life in kidney transplant recipients (KTRs) continues to remain inferior to those without chronic kidney disease (CKD) [4]. To reduce the risk of long-term complications associated with transplantation, including graft failure, KTRs are encouraged to engage with their healthcare and take greater responsibility for their health [5]; this is termed self-management [6]. KTRs who engage in self-management behaviours have improved clinical markers and report better medication adherence [7]. Self-management comprises of three core tasks: medical, role, and emotional management [8]. For KTRs, medical management includes adherence to complex medication regimens, self-assessment for symptoms and complications, attending appointments and cancer screening, and avoidance of unwell individuals [5, 9]. Emotional management describes dealing with the negative emotions of their condition such as fear of graft failure. Role management includes social and behavioural adaptations, such as eating a balanced diet, maintaining hydration and regular physical activity [9].

Engagement in self-management behaviours in KTRs remain poor, with studies reporting low levels of physical activity [10], inconsistent dietary adherence [11], and high levels of medication non-adherence [12]. Understanding the facilitators and barriers towards effective self-management can aid in the development of targeted resources to support these behaviours. A qualitative systematic review of motivations, challenges, and attitudes in KTRs identified themes relating to the overarching perspectives of self-management such as burdensome treatment, responsibilities, and empowerment through autonomy [9]. Despite synthesising fifty studies, many included only explored specific experiences relating to single aspect of self-management (e.g., medication adherence, stressors and coping strategies, and post-operative recovery) [13–16]. Other studies exploring self-management in KTRs have excluded key groups such as individuals less than one-year post-transplantation [17], a population of key importance due to their vulnerability to graft loss and death [18]. Research has also focused on specific age groups such as adolescents [19] or older KTRs [20]. Been-Dahmen et al. [21] explored the self-management challenges and support needs of KTRs and utilised the three self-management tasks described by Corbin and Strauss [8] when both designing and reporting their study. However, due to being conducted at a single centre in the Netherlands, and subsequently having a majority Dutch sample (81.3%), it is important to understand how their findings may transfer to other KTR populations.

To date, no studies have reported KTRs’ experiences alongside an assessment to quantify individuals perceived self-management ability. The ‘Patient Activation Measure’ (PAM) assesses individuals’ perceived ability to manage their own health and care [22]. The important role of the PAM in understanding self-management behaviours in KTRs has been recognized in the United Kingdom (UK) [23, 24], and was recommended to inform kidney disease policy in the United States [25]. A recent report suggests that the PAM could be pivotal when developing patient-centred self-management interventions for KTRs, and could inform interview topics and facilitate the representation of the experiences of those with different self-management abilities [25].

With self-management tasks changing considerably post-transplantation, there is a need to understand the overall experiences, facilitators, and barriers of self-management to inform healthcare professionals (HCPs) on how to deliver effective tailored support to KTRs. The purpose of this study was to explore the facilitators and barriers towards self-management in a UK KTR population.

## Materials and methods

### Study design and participants

This is a qualitative sub-study of DIMENSION-KD (ISRCTN84422148), a national multi-site prospective observational research study. Participants were recruited from routine outpatient clinics at the Leicester General Hospital, UK. For this study, the inclusion criteria were ≥ 18 years of age, a functioning kidney transplant, and the ability to provide written informed consent. Exclusion criteria were CKD Stages 1–5 without transplantation.

The aim was to recruit 12 participants; this sample size was chosen to reflect sufficient diversity in views and experiences within the available time and resources. This number reflected guidelines created to ensure an appropriate sample size [26], and was subject to change throughout the interview process as it was dependant on the richness of data derived from individual participants [27]. In order to capture diversity in responses, participants were recruited using purposive sampling, utilising maximum variation sampling to ensure a representative population with a range of characteristics [28].

Those invited to be interviewed were contacted by phone or email, using details provided by participants, to arrange a suitable time for interview. Ethical approval was provided by the East-Midlands Leicester Central Research Ethics Committee (18/EM/0117). Written informed consent was obtained prior to the commencement of the interview. Transcripts were anonymised and IDs were given to participants.

Consent was obtained to access clinical data (eGFR, years since receiving transplant) from medical notes and questionnaire results (age, ethnicity, employment status, PAM score) from a different part of the research study. Patient activation was assessed by the PAM—where individuals answered thirteen statements relating to health behaviours on a Likert scale (disagree strongly, disagree, agree, agree strongly or N/A). PAM scores between 0 and 100 were calculated [22, 29] to categorise participants into one of four activation levels; Level 1 (< 47.0, lowest), Level 2 (47.1–55.1), Level 3 (55.2–67.0), Level 4 (> 67.0, highest) [22, 30]. PAM has been validated and recommended for use in KTRs [31].

### Interview procedure

Individual semi-structured interviews were used to explore the perceptions and experiences of self-management. An interview schedule was developed through familiarisation with current literature, exploring the three self-management tasks described by Corbin and Strauss [8], and also reflecting core statements within the PAM (skills, knowledge and confidence). A renal pharmacist provided insights into medication regimens, and two KTRs provided further feedback on the types of self-management tasks to be discussed. Minor revisions were made prior to conducting interviews to reflect topics that emerged (e.g., perceived role of family members in KTRs’ care). The schedule was pilot tested with a KTR to ensure that the interview schedule was appropriate, easily understood, and functional. As the pilot interview was data-rich, it was subsequently included in the final analysis.

Interviews were conducted face-to-face within a quiet meeting room in the research department, between October 2019 and February 2020, by a medical student (KEM) completing a master’s project with the research team. An experienced qualitative researcher (CJL) observed two interviews. Participants had no previous relationship with the researchers prior to study commencement. KEM kept a personal reflective logbook during interview conduction and analysis. Interviews were audio-recorded and transcribed verbatim by a professional transcription company.

### Qualitative analysis

QSR International’s NVivo9 software was used to manage the data, which were analysed using reflexive thematic analysis, described by Braun and Clarke [32, 33]. KEM familiarised themself with the complete data set through listening to audio-recordings and annotating transcripts, and independently identified initial codes using an inductive approach. A sample of transcripts were independently coded by CJL. Following confirmation of similar code derivation, the remaining transcripts were coded by KEM. Potential themes were created through identifying relationships between codes, re-focusing and collating them to form over-arching concepts. CJL refined themes with KEM throughout this process, and definitions of themes were agreed. It was recognised that emerging themes were strongly linked to the three self-management tasks by Corbin and Strauss [8], and was likely reflective of the tasks being embedded within the interview schedule. Thus, to provide structured demonstration of findings, themes were mapped onto these tasks, and relevant quotes were selected to illustrate the findings. The COREQ guidelines were followed when reporting the methods.

## Findings

### Summary of participant characteristics

Twenty-nine KTRs were invited to participate, 18 of whom responded: two declined to take part and two were uncontactable. Further recruitment ceased due to the Covid-19 pandemic. Thus, a total of 11 interviews were conducted. Participant characteristics are displayed in Table 1. Interviewees comprised of eight males and three females, with a mean age of 55 (range 33–77) years. The mean eGFR was 48.0 (± 21.2) mL/min/1.73 m^2^. PAM scores ranged from 51.0 to 100 (Levels 2–4).

**Table 1:** Participant characteristics

No	Gender	Age (years)	Ethnicity	Employment	Years since receiving transplant	eGFR (mL/min/1.73 m^2^)	PAM Score	PAM Level
1	Female	33	Caucasian	Employed	7	58	72.5	4
2	Male	61	Indian	Employed	27	32	^a^	^a^
3	Male	38	Caucasian	Employed	6	30	65.5	3
4	Male	68	Caucasian	Retired	2	58	100	4
5	Male	64	Caucasian	Retired	< 1^b^	51	65.5	3
6	Male	77	Caucasian	Employed	6	18	53.2	2
7	Male	33	Indian	Employed	< 1	50	^a^	^a^
8	Male	59	Caucasian	Employed	10	67	55.6	3
9	Female	49	Caucasian	Unemployed	< 1	88	65.5	3
10	Female	59	Caucasian	Employed	10	37	51	2
11	Male	59	Other	Employed	< 1	42	67.8	3

*PAM* Patient Activation Measure

^a^Data not provided by participant

^b^Participant had received more than one transplant

Interview duration ranged from 48 to 86 min (mean 68 min). Participants described their role in self-managing their health and kidney transplant. Eight themes were identified and, to both provide a structured display of findings and reflect the integral role of these tasks in exploring our research question, they were mapped onto their relevant task described by Corbin and Strauss [8]. Interviews prioritised exploring motivators to self-management and so more facilitators than barriers were identified. Themes present both barriers and facilitators within them and are displayed with relevant quotes in tables relating to their associated self-management task.

### Task 1: Medical management

#### *Exemplar quotes are presented in Table **2*

**Table 2:** Themes applied to the medical management task described by Corbin and Strauss (1988)

Theme	Minor themes	Exemplar quotes from kidney transplant recipients
Gathering health-related knowledge	Information collecting	*“I just pick it up on the way through, various consultants and doctors, listen to people … that’s how you learn” (Male, age 77, PAM Level 2)* *“… [exercise] was something that I was excited about but also a little daunted about because I'm like well what can I do … will that cause any damage?”* *(Male, age 33, PAM Level 3)* *“I believe that a better understanding of your condition, of all the aspects, leads to, well better treatment or, at the end of the day, lifestyle.”* *(Male, age 64, PAM Level 3)*
	Reputable sources	*“There’s a lot of rubbish about certain subjects online …” (Male, age 77, PAM Level 2)* *“… it’s my own accord now, so in terms of the diet, I’ve spoken to the dietician who’s given me his input.”* *(Male, age 33, PAM not reported)*
	Information as a source of anxiety	*“… I never once have read the [medication] leaflet that you get inside because I know there’s that many side effects, I just don't want to know. And I just, I’d rather be, like, just completely oblivious to it.” (Female, age 33, PAM Level 4)*
Complexity of required treatment	Symptoms	*“I don’t exercise very much, because even now I get breathless … It stops me being mobile, because I can walk for so far and then stop, where I’ve got to.“ (Male, age 77, PAM Level 2)* *“I would say I am still in recovery now. You do certain things and you get twinges; you get a lot of infections; you get a lot of other stuff.” (Female, age 49, PAM Level 3)*
	Complications	*“The last time I got sick was just before Christmas … I was in hospital for a month, and then I was probably still off work for about a month or a month and a half after I got out … I just rested.” (Female, age 59, PAM Level 2)* *“… I was just so poorly and I just got one thing after the other … I ended up with urosepsis and I was in hospital, like, in here for a week and my kidney function halved and it was just awful.” (Female, age 33, PAM Level 4)*
	Specialist support	***“*** *[GPs] give you ten minutes … they only want one problem, you can't say [a] second one, ‘OK come next time’, I'm there, why not just sort it out. Because I've got so many problems because it might be related to the other one…” (Male, age 61, PAM not reported)* *“I’d rather contact them here than my own doctors … because they are the experts on the kidneys.” (Male, age 68, PAM Level 4)*

##### Theme 1: Gathering health-related knowledge

Participants believed that having knowledge of their condition and an awareness of how to self-manage increased their confidence to undertake self-management tasks. Self-management knowledge was considered to increase with the progression of time since their transplant, as a result of engaging more in their healthcare. The desired amount of information to effectively self-manage varied, with some considering excess knowledge as a source of anxiety, and others wanted more.

##### Theme 2: Complexity of required treatment

The combined burden of symptoms, medication side effects, underlying kidney condition, and comorbidities were considered to limit engagement with self-management. Participants with multiple conditions believed that General Practitioners (GPs) had insufficient time to support each condition effectively. Complications such as infections were described as disabling, with long hospital stays resulting in negative impact on mental health and exacerbated symptom burden. Fatigue was considered as a debilitating symptom which limited activities and could only be relieved by resting. Some participants reported being unable to complete daily tasks or maintain employment due to their symptom burden.

### Task 2: Role management

#### *Exemplar quotes are presented in Table **3*

**Table 3:** Themes applied to the *role management* task described by Corbin and Strauss (1988)

Theme	Minor themes	Exemplar quotes from kidney transplant recipients
Building relationships with healthcare professionals	Continuity of care	*“You’ve got the consistency with the doctors that you see, and they always explain everything very well … I think probably because it’s been with me for so long, and if I’ve got a problem, I know I can always come here.”* *(Female, age 59, PAM Level 2)* *“Continuity is important … being known by the team, there is the personal aspect, they know you. I also know them.”* *(Male, age 64, PAM Level 3)*
	Collaborative care	*“When I see [Doctor] on a roughly three, four monthly basis, I bring that up during the discussions … we cut that out and try and find something alternative etcetera.”* *(Male, age 64, PAM Level 3)* *“They wanted to give me more medication … I just said look, can you give me some time … to see whether I can bring it [blood pressure] down myself through meditation or anything like that, and yeah, they were fine with it.”* *(Male, age 33, PAM not reported)*
	Ease of access	*“If I was abroad, I can e-mail [Doctor]’s secretary and say I’ve got this or that … they’re at the end of the phone if something is not quite right.”* *(Male, age 68, PAM Level 4)*
	Empowerment	*“[A] lot of them have said this to me, you’ve got your whole life ahead of you, so just be on top of it.”* *(Male, age 33, PAM not reported)* *“They’ve always said, ‘you are the person that knows more about your health than anyone else because you're living with it’.”* *(Female, age 33, PAM Level 4)*
Creating routines within daily life	Creating habits	*“[Taking medication] becomes a habit. First thing in the morning, last thing at night.”* *(Male, age 77, PAM Level 2)* ***“****I’ve got an alarm for my tablets … put diary dates in when I need to order my tablets as well…”* *(Male, age 68, PAM Level 4)* *“… a two and a half mile walk on an either early evening or whenever, even at night … that forms a part of our lifestyle*.” *(Male, age 64, PAM Level 3)*
	Preparing for disruptions	*“[If] I'm going out or going out for a meal, I’ll put a reminder on my phone [to take medications].”* *(Male, age 38, PAM Level 3)* *“The only times it gets a bit [difficult] is if you go out of your routine … I might take them [medications] a little bit early before I go, or other times I might just take them with me.” (Female, age 59, PAM Level 2)*
Setting goals and identifying motivators	Goals	*“[Weight loss] restricts my life quite a lot … It’s not like a never-ending thing. I’ve got a goal” (Male, age 59, PAM Level 3)* *“Because of the nature of my work as well, you had to be physically fit” (Male, age 38, PAM Level 3)*
	Motivators	*“I'm thinking about [daughter], she makes me stronger… why worry about me.” (Male, age 61, PAM not reported)* *“I’m glad that I’ve got a job, because it gives me something to do … at the weekend it’s a devil of a job to do anything, because I’ve not got that objective to get to.” (Male, age 77, PAM Level 2)* *“The transplant has been a very precious gift … I want to try and do everything I can to try and maintain it” (Male, age 59, PAM Level 3)*

##### Theme 3: Building relationships with healthcare professionals

Participants believed that establishing relationships with HCPs helped build trust, supported them to feel involved with healthcare decisions and enabled the delivery of tailored advice. These relationships were formed through collaborative consultations with HCPs and continuity of clinical support. Ease of access to medical support was considered to be reassuring to participants, and HCPs who took responsibility for their patients, answered questions, provided manageable recommendations, and empowered patients were perceived to offer good quality care.

##### Theme 4: Creating routines within daily life

Medication, dietary, and exercise routines were perceived to facilitate self-management by integrating tasks into daily life. Most participants described adjusting medication routines around meals or work schedules, with some participants utilising dosette boxes and alarms as reminders; preparing for routine disruptions, such as holidays or life-events, were considered to ensure consistency.

##### Theme 5: Setting goals and identifying motivators

Goals were reported to motivate participants to self-manage, and included weight loss, exercise consistency, and high fluid intake, which participants tracked themselves or by using apps. Whilst some participants felt discouraged when they were not achieved and believed that goals restricted their lifestyles, many discussed how they provided an endpoint to persevere towards. Family members, career aspirations, inspiring stories from other KTRs, and aspiring for a long graft lifespan were considered motivators to engage in self-management behaviours.

### Task 3: Emotional management

#### *Exemplar quotes are presented in Table **4*

**Table 4:** Themes applied to the *emotional management* task described by Corbin and Strauss [8]

Theme	Minor themes	Exemplar quotes from kidney transplant recipients
Adjusting to a new health status	Emotional impact	*“… it’s almost like a PTSD thing [after transplantation], whoa, what the hell have I just been through over the last two, three years? Whoa, suddenly hits you …” (* *Female, age 49, PAM Level 3)* *“… when I got home [after transplantation], oh I was a nightmare … it’s just such a massive thing that you’ve gone through … the enormity of everything that’s gone on hits you” (Female, age 33, PAM Level 4)*
	New limits	*“… [after transplantation] I genuinely felt as though I had enough energy to run a marathon … Mentally I could do it, but with my body and stuff I couldn’t.”* *(Male, age 33, PAM not reported)* *“… you restrict yourself [on dialysis] … then when you come off that, they tell you to drink a lot of water, you mentally can’t get round it.”* *(Male, age 77, PAM Level 2)*
	Perceived vulnerability	*“… those early days you’re very very, feel very vulnerable, not very confident at all … I can remember going out for the first time with my friends, and this pub was really busy, and I thought I didn’t want to stand too close to them, I thought because if I end up with a fight and you get punched in this area.”* *(Male, age 68, PAM Level 4)* *“I'm so much more anxious about, like, something happening to my kidney … I'm so paranoid about something happening and getting that ill again, definitely.”* *(Female, age 33, PAM Level 4)*
Establishing peer support	Sharing of experiences	***“*** *I've got quite a few friends that have had transplants … it’s so good to talk to them because they fully understand how you feel, and we can support each other.” (Female, age 33, PAM Level 4)* *“Knowing what I know now, I would like to pass that information to someone, even my age or younger … just tell them about the diet, medication …” (Male, age 33, PAM not reported)*
	Limitations of peer support	*“I found that reading online forums about holidays when I was dialysing depressed me.” (Male, age 77, PAM Level 2)* *“If there was a patient who could explain, that’s fine, but I think if I had an issue, a complication or a problem I would probably just speak to the health professional.” (Male, age 59, PAM Level 3)*
Support from family and friends	Partnership with family and friends	***“*** *My wife … she’s genuinely given me more knowledge, she’s assisting me with the diet and stuff, she comes to my appointments … she would ask more things, so then it got me thinking about what I should be asking.” (Male, age 33, PAM not reported)* *“All my friends are really good … if I was supposed to be meeting them and they don't feel well they’ll text me and they’ll go ‘look, just to let you know, like, I think I'm coming down with a cold’ or whatever and we will rearrange.” (Female, age 33, PAM Level 4)*
	Educating support networks	***“*** *They should say well I would suggest you come and bring your wife along … we all have a different view or angle of what was said. And in that way, on the way home people can discuss, well what do you think was said etcetera.” (Male, age 64, Pam Level 3)* *“I don’t think [Husband] or [Daughter] have ever been told that if you want to give us a ring we can sit down and we can explain stuff to you. So that would be helpful.” (Female, age 49, PAM Level 3)*

##### Theme 6: Adjusting to a new health status

Most participants discussed the emotional impact of receiving a transplant and their fears of graft loss. Managing complex health recommendations and adjusting to reduced restrictions in the early post-transplant phase was considered to be overwhelming, impacting upon participants’ mental health. Nearly all participants described feeling vulnerable and anxious due to missed medications or complications. Participants were fearful of harming their graft, especially when exercising, being around unwell individuals, or in crowds of people.

##### Theme 7: Support from family and friends

Almost all participants expressed a desire for family and friends to receive education on their condition, the emotional impact of receiving a transplant, and how to assist with self-management behaviours. Many participants believed that having family members attend their appointments helped them to understand their condition better, provided appropriate emotional support, and supported participants to engage more with their health.

##### Theme 8: Establishing peer support networks

A small number of participants explained that they avoided seeking peer support because they previously encountered scaremongering and negativity. Nevertheless, the majority of participants described experiencing a ‘community’ when engaging with other KTRs and they suggested that providing support groups, online forums, and patient information days could emotionally support KTRs.

## Discussion

In this study, we report the facilitators and barriers to self-management in KTRs. This information is key to understanding how to improve self-management behaviours in individuals. Through reporting participant PAM levels, our study provides further information on each individual’s perceived self-management abilities to complement the understanding gained from their lived experiences. Our findings demonstrate that effective self-management requires support to complete each of the three self-management tasks described by Corbin and Strauss [8]: medical, role, and emotional management. Gathering sufficient information on how to self-manage was described as meaningful to undertake medical management, whilst individual’s symptom burden, complications, and comorbidities were considered barriers. Role management was facilitated by establishing relationships with HCPs, building routines, setting goals, identifying motivators, and integrating both peer and family support networks into individuals’ healthcare. Emotional management was considered to be adversely impacted by the emotional burden of transplantation, including fear of graft failure and individuals’ perceived vulnerability.

Establishing relationships with HCPs was facilitated through collaborative consultations, continuity of care, and empowerment from HCPs and appeared to influence individuals’ health-related knowledge. The importance of developing partnerships, shared decision-making, and desired qualities of HCPs has been discussed within other KTR populations [21, 34]. Active interactions with HCPs can empower individuals and nurture intrinsic motivation, defined as the completion of tasks for personal satisfaction [35]. With intrinsic motivation being a prominent motivator for behavioural change [36], HCPs should actively listen to their patients’ descriptions of their lifestyles and integrate recommendations based on their desired outcomes. These consultations could involve the creation of action plans which identify realistic and tailored health targets [35, 37] to increase recipients’ self-efficacy (an individual’s confidence and beliefs in their capabilities to complete tasks) [38].

Participants discussed the importance of identifying motivators to engage with self-management and setting health-related goals. The significance of KTRs setting goals to achieve weight loss has been previously mentioned [39]; however, the motivating factor for participants to reach targets varied: some strived for looser fitting clothes whilst others were motivated by weight reductions on scales. Our study similarly found that motivators were diverse and influenced by previous experiences, circumstances, and beliefs. Individualised techniques to promote self-efficacy, including motivational interviewing or developing action plans, could influence self-management behaviours in KTRs [40]. Whilst priorities can differ with donor type, with individuals receiving a live donation feeling obligated to care for themselves and those who receive a deceased donation can be motivated by guilt [9], this was not discussed by our participants. Further research may be required to explore differences in self-management behaviours between those who receive a live donation, both related and unrelated, and those who receive a deceased donation.

The routinising of diet, exercise, and medication regimens were perceived to facilitate consistent engagement with self-management. Similar routines have been discussed in other KTR populations, particularly relating to medication regimens [16, 17, 41, 42]. Like other studies [16, 41, 42], our participants emphasised that their routines are dynamic and prone to change with holidays or life-events, and so they utilise reminders (e.g., alarms or taking medications earlier/later) to overcome potential disruptions. Tailored recommendations based on individuals’ lifestyles and promote self-regulation of adherence behaviours to promote consistent self-management [43].

Social support networks were considered to encourage participants to actively engage during consultations and supported healthy eating and exercise. Family support can promote healthy behaviours [17], and engaging the family within KTRs’ care could be further prioritised by providing education on supporting self-management tasks and exploring the concerns of the family. Social support should be integrated with consideration as disparities in their perceived illness states means some KTRs have experienced limited emotional support from relatives [34]. Similar to other studies, peer support was not universally desired by participants [21], and some avoided due to ‘scaremongering’ [44]. However, talking to other KTRs can validate concerns and facilitate the sharing of knowledge. Reviews of one-to-one peer-led support in CKD patients received positive acclaim with interactions described as providing hope, reassurance and encouragement [45]. Such peer-led programmes or larger support groups could be integrated into KTRs’ care pathways, with a focus on managing the emotional barriers experienced, including how to adjust from prior restrictions, worries about engaging with exercise, coping with anxiety-provoking situations, and how to overcome negative emotions.

Fatigue, breathlessness, and pain were prevalent amongst participants and were considered to limit their ability to engage in self-management tasks and daily activities. Symptom burden has similarly reduced physical function in other KTR populations [39, 46], with older-aged KTRs feeling frustrated when complications, comorbidities, and reduced physical function persist following transplantation [20]. The emotional and physical burden of these factors may influence KTRs’ abilities to self-manage, therefore it remains imperative to understand the individuals’ perceived treatment burden. Routine assessment of patients’ symptoms and comorbidities could inform tailored education of their management.

Managing the prevalent and complex emotional impact of transplantation warrants more attention. Our participants described feeling overwhelmed and vulnerable post-transplantation, experiencing fear when engaging in daily activities. Heightened emotional responses immediately following transplantation [15, 44, 47], and distress following potential changes or complications post-transplantation [9] have previously been reported. Guidance and reassurance on how to safely re-engage in self-management activities (e.g., their bodies’ physical limitations and activity recommendations), and signposting to psychological interventions [14] could support KTRs.

The main limitation is that interviews were conducted at one-time point. Self-management behaviours are likely to change over time so responses may change depending on factors such as time since transplant or complications. Some participants had recently received a transplant; therefore, their self-management may not have been as well-established as other interviewees. This study was conducted as part of a master’s project with data collection limitations further impeded by the COVID-19 pandemic. Nevertheless, data were rich in experiences and analysis reflected a thorough exploration of the research question. As proposed by Braun and Clarke [48], we provide detailed descriptors of our analysis process as an alternative to commenting on data saturation, due to the concept not aligning with our reflexive thematic analysis stance. Additionally, we recognise the limitations of transferability with a majority male and Caucasian sample; however, the samples were a good reflection of the larger DIMENSION-KD study (*n* = 743) (our study 73% versus 68% male; 73% Caucasian versus 94% White British) [49]. Further efforts to explore experiences of other ethnic groups is needed. In addition, exploring the experiences of socioeconomically disadvantaged individuals, older individuals, and paediatric KTRs would merit consideration in future research.

## Conclusion

More facilitators than barriers to self-management were reported by KTRs. This study demonstrates the importance of understanding lived experiences and perceived needs to tailor self-management support. KTRs experience facilitators and barriers in all aspects of self-management indicating that that holistic care should address all self-management components; medical, role, and emotional management. Greater focus should be given to support role and emotional management in this population.

## References

[1] Tonelli M, Wiebe N, Knoll G (2011). Systematic review: kidney transplantation compared with dialysis in clinically relevant outcomes. Am J Transpl.

[2] Purnell TS, Auguste P, Crews DC (2013). Comparison of life participation activities among adults treated by hemodialysis, peritoneal dialysis, and kidney transplantation: a systematic review. Am J Kidney Dis.

[3] Kaballo MA, Canney M, O'Kelly P, Willians Y, O'Seaghdha CM, Conlon PJ (2018). A comparative analysis of survival of patients on dialysis and after kidney transplantation. Clin Kidney J.

[4] Hernandez Sanchez S, Carrero JJ, Garcia Lopez D, Herrero Alonso JA, Menendez Alegre H, Ruiz JR (2016). Fitness and quality of life in kidney transplant recipients: case-control study. Med Clin (Barc).

[5] Gordon EJ, Prohaska T, Siminoff LA, Minich PJ, Sehgal AR (2005). Can focusing on self-care reduce disparities in kidney transplantation outcomes?. Am J Kidney Dis.

[6] Lorig KR, Holman H (2003). Self-management education: history, definition, outcomes, and mechanisms. Ann Behav Med.

[7] Ganjali R, Khoshrounejad F, Mazaheri Habibi MR (2019). Effect and features of information technology-based interventions on self-management in adolescent and young adult kidney transplant recipients: a systematic review. Adolesc Health Med Ther.

[8] Corbin JM, Strauss A (1988). Unending work and care: managing chronic illness at home.

[9] Jamieson NJ, Hanson CS, Josephson MA (2016). Motivations, challenges, and attitudes to self-management in kidney transplant recipients: a systematic review of qualitative studies. Am J Kidney Dis.

[10] Gustaw T, Schoo E, Barbalinardo C (2017). Physical activity in solid organ transplant recipients: participation, predictors, barriers, and facilitators. Clin Transpl.

[11] Nolte Fong JV, Moore LW (2018). Nutrition trends in kidney transplant recipients: the importance of dietary monitoring and need for evidence-based recommendations. Front Med (Lausanne).

[12] Cossart AR, Staatz CE, Campbell SB, Isbel NM, Cottrell WN (2019). Investigating barriers to immunosuppressant medication adherence in renal transplant patients. Nephrology (Carlton).

[13] Amerena P, Wallace P (2009). Psychological experiences of renal transplant patients: a qualitative analysis. Couns Psychother Res.

[14] Baines LS, Joseph JT, Jindal RM (2002). Emotional issues after kidney transplantation: a prospective psychotherapeutic study. Clin Transpl.

[15] Gill P (2012). Stressors and coping mechanisms in live-related renal transplantation. J Clin Nurs.

[16] Orr A, Orr D, Willis S, Holmes M, Britton P (2007). Patient perceptions of factors influencing adherence to medication following kidney transplant. Psychol Health Med.

[17] Ndemera H, Bhengu BR (2017). Motivators and barriers to self-management among kidney transplant recipients in selected state hospitals in South Africa: a qualitative study. Health Sci J.

[18] Farrugia D, Cheshire J, Begaj I, Khosla S, Ray D, Sharif A (2014). Death within the first year after kidney transplantation: an observational cohort study. Transpl Int.

[19] Tong A, Morton R, Howard K, McTaggart S, Craig JC (2011). "When I had my transplant, I became normal". Adolescent perspectives on life after kidney transplantation. Pediatr Transpl.

[20] Pinter J, Hanson CS, Chapman JR (2017). Perspectives of older kidney transplant recipients on kidney transplantation. Clin J Am Soc Nephrol.

[21] Been-Dahmen J, Grijpma J, Ista E (2018). Self-management challenges and support needs among kidney transplant recipients: a qualitative study. J Adv Nurs.

[22] Hibbard JH, Mahoney ER, Stockard J, Tusler M (2005). Development and testing of a short form of the Patient Activation Measure. Health Serv Res.

[23] NHS England, Public Health England, Monitor, Health Education England, Care Quality Commission, NHS Trust Development Authority. *NHS five year forward view.* 2014. https://www.england.nhs.uk/wp-content/uploads/2014/10/5yfv-web.pdf. Accessed 26 Jan 2021

[24] Gair RM, Stannard C, Wong E, et al. *Transforming participation in chronic kidney disease: programme report.* 2019. https://www.thinkkidneys.nhs.uk/ckd/wp-content/uploads/sites/4/2019/01/Transforming-Participation-in-Chronic-Kidney-Disease-1.pdf. Accessed 26 Jan 2021

[25] Nair D, Cavanaugh KL (2020). Measuring patient activation as part of kidney disease policy: are we there yet?. J Am Soc Nephrol.

[26] Francis JJ, Johnston M, Robertson C (2010). What is an adequate sample size? Operationalising data saturation for theory-based interview studies. Psychol Health.

[27] Saunders B, Sim J, Kingstone T (2018). Saturation in qualitative research: exploring its conceptualization and operationalization. Qual Quant.

[28] Etikan I, Musa SA, Sunusi RA (2016). Comparison of convenience sampling and purposive sampling. Am J Theor Appl Stat.

[29] Hibbard JH, Greene J (2013). What the evidence shows about patient activation: better health outcomes and care experiences; fewer data on costs. Health Aff (Millwood).

[30] Hibbard JH, Mahoney ER, Stock R, Tusler M (2007). Do Increases in patient activation result in improved self-management behaviors?. Health Serv Res.

[31] Lightfoot CJ, Wilkinson TJ, Memory KE, Palmer J, Smith AC (2021). Reliability and validity of the patient activation measure in kidney disease: results of rasch analysis. Clin J Am Soc Nephrol.

[32] Braun V, Clarke V (2006). Using thematic analysis in psychology. Qual Res Psychol.

[33] Braun V, Clarke V (2019). Reflecting on reflexive thematic analysis. Qual Red Sport Exerc Health.

[34] Schmid-Mohler G, Schäfer-Keller P, Frei A, Fehr T, Spirig R (2014). A mixed-method study to explore patients' perspective of self-management tasks in the early phase after kidney transplant. Prog Transplant.

[35] Bodenheimer T, Lorig K, Holman H, Grumbach K (2002). Patient self-management of chronic disease in primary care. JAMA.

[36] Ryan RM, Deci EL (2000). Intrinsic and extrinsic motivations: classic definitions and new directions. Contemp Educ Psychol.

[37] Thomas-Hawkins C, Zazworsky D (2005). Self-management of chronic kidney disease. Am J Nurs.

[38] Bandura A (1991). Social cognitive theory of self-regulation. Organ Behav Hum Decis Process.

[39] Stanfill A, Bloodworth R, Cashion A (2012). Lessons learned: experiences of gaining weight by kidney transplant recipients. Prog Transpl.

[40] Weng LC, Dai YT, Huang HL, Chiang YJ (2010). Self-efficacy, self-care behaviours and quality of life of kidney transplant recipients. J Adv Nurs.

[41] Gordon EJ, Gallant M, Sehgal AR, Conti D, Siminoff L (2009). Medication-taking among adult renal transplant recipients: barriers and strategies. Transpl Int.

[42] Ruppar TM, Russell CL (2009). Medication adherence in successful kidney transplant recipients. Prog Transpl.

[43] Low JK, Williams A, Manias E, Crawford K (2014). Interventions to improve medication adherence in adult kidney transplant recipients: a systematic review. Nephrol Dial Transpl.

[44] Urstad KH, Wahl AK, Andersen MH, Øyen O, Fagermoen MS (2012). Renal recipients’ educational experiences in the early post-operative phase – a qualitative study. Scand J Caring Sci.

[45] Hughes J, Wood E, Smith G (2009). Exploring kidney patients' experiences of receiving individual peer support. Health Expect.

[46] Gordon EJ, Prohaska TR, Gallant M, Siminoff LA (2009). Self-care strategies and barriers among kidney transplant recipients: a qualitative study. Chronic Illn.

[47] Schipper K, Abma TA, Koops C, Bakker I, Sanderman R, Schroevers MJ (2014). Sweet and sour after renal transplantation: a qualitative study about the positive and negative consequences of renal transplantation. Br J Health Psychol.

[48] Braun V, Clarke V (2021). To saturate or not to saturate? Questioning data saturation as a useful concept for thematic analysis and sample-size rationales. Qual Res Sport Exerc Health.

[49] Wilkinson TJ, Memory K, Lightfoot CJ, Palmer J, Smith AC (2021). Determinants of patient activation and its association with cardiovascular disease risk in chronic kidney disease: a cross-sectional study [published online ahead of print April 9, 2021]. Health Expect.

